# Increased ethanol consumption after interruption of fat bingeing

**DOI:** 10.1371/journal.pone.0194431

**Published:** 2018-03-28

**Authors:** M. Carmen Blanco-Gandía, José Miñarro, Maria Asuncion Aguilar, Marta Rodríguez-Arias

**Affiliations:** Unidad de Investigación Psicobiología de las Drogodependencias, Departamento de Psicobiología, Facultad de Psicología, Universitat de València, Valencia, Spain; Oregon Health and Science University, UNITED STATES

## Abstract

There is a marked comorbidity between alcohol abuse and eating disorders, especially in the young population. We have previously reported that bingeing on fat during adolescence increases the rewarding effects of ethanol (EtOH). The aim of the present work was to study if vulnerability to EtOH persists after cessation of binge eating. OF1 mice binged on fat (HFB: high-fat binge) during adolescence (PND 25–43) and were tested for 15 days after the last access to HFB (on PND 59) using the self-administration paradigm, the conditioned place preference (CPP) and locomotor sensitization to ethanol. Our results showed that after 15 days of cessation of fat ingestion, mice increased their consumption of ethanol and showed greater motivation to obtain ethanol. On the other hand, no effects were observed in the CPP, while an increased locomotor response to ethanol was detected. The present results confirm and extend our previous study demonstrating that the compulsive intake of fat induces long-lasting effects on the reward system that lead to an increased consumption of EtOH.

## Introduction

Adolescence is a developmental period of elevated synaptic plasticity [1, 2] in which individuals become especially vulnerable to environmental threats, such as stress, drug abuse or inadequate dietary habits [3–5]. In this period, there is an imbalance between an increased sensitivity to motivational cues and the still-maturing inhibitory control system (delayed maturation of the prefrontal cortex), which leads to a heightened activation of reward-relevant regions [6]. Among the factors that contribute to increased vulnerability to drug use, dietary conditions might play a greater role than previously thought [1, 7, 8]. Currently, there is an increasingly prevalent high-fat, “fast-food” culture with rising rates of obesity in developed countries, particularly among adolescents [7, 9, 10]. Behavioral sensitivity to rewards is believed to peak during adolescence and then gradually decline during adulthood [11, 12]. It is important to remember that adolescents are more prone than adults to developing eating disorders, such as anorexia, bulimia and binge eating [13]. For example, adolescent rats exhibit the greatest caloric intake relative to their bodyweight throughout their lifespan [14]. Likewise, adolescent humans exhibit developmental hyperphagia and elevated metabolic activity [15].

Alcohol is one of the first drugs of choice among teenagers [16]. Substance abuse in early stages of life is linked with a higher rate of drug abuse and dependence in adulthood [17, 18]. In this period of brain maturation alcohol can have a negative impact on its structure and function [19], producing short and long-term consequences such as memory impairment and neural cell death in several brain regions [20], which are mostly irreversible [21].

Drugs and hedonic eating share common dopaminergic pathways, and a great number of studies have demonstrated the comorbidity that exists between them [22–24]. It is known that eating for pleasure, and not for metabolic need, affects dopamine release and the neural pathways that are involved in reward and motivation processes, which in turn further reinforces this type of eating behavior [10, 25, 26]. Particularly, binge-eating is considered a specific form of overeating that in recent years has been studied and deliberated as an addictive behavior that mimics that of drugs of abuse [27]. It is characterized by a dysfunctional appetite, which is manifested by an intermittent, excessive intake of caloric food. Many teenagers exhibit this kind of hedonic eating [28], which includes eating for pleasure rather than for metabolic need, without meeting the clinical criteria for a binge-eating disorder. Moreover, studies in animals have confirmed that adolescent rats are more prone to binge-eating than adults [29]. Nevertheless, it has been proposed that binge-eating, as a maladaptive behavior, could work as a gateway for the development of drug addiction [30–33]. We have recently confirmed that bingeing on fat increases cocaine and EtOH consumption and the conditioned rewarding effects of both drugs [32, 33]. First, we demonstrated that animals bingeing on fat were more sensitive to the reinforcing effects of a subthreshold dose of cocaine in the conditioned place preference and presented enhanced cocaine self-administration. Moreover, after a period of withdrawal, those animals that binged on fat exhibited reinstatement of drug-seeking in the self-administration task [32]. In another study, we confirmed that these animals were more sensitive to the conditioned rewarding effects of subthreshold doses of ethanol and presented greater ethanol consumption [33]. In both studies, animals displayed several changes in gene expression involving the opioid, cannabinoid and ghrelin systems. Similar results have been obtained with sugar; Avena and co-workers [34] reported that rats that binged intermittently on sugar consumed more EtOH (9%) than those with *ad libitum* access to sugar or chow. These results point to a co-morbidity between binge-eating disorders and alcohol intake.

There is extensive evidence of dependence on sugar and withdrawal [35–40]. For example, rats allowed intermittent access to sugar and then forced to abstain exhibit enhanced intake of alcohol [34]. Data regarding the dependence of high-fat food, on the other hand, are scarce. We have previously reported that 2 weeks after the sudden interruption of continuous access to fat, animals present higher anxiety levels, thus confirming a state of withdrawal [37]. In addition, the mice in question were more sensitive to the conditioned rewarding effects of cocaine. With respect to the consequences of abrupt cessation of fat bingeing, we observed an increase in anxiety 15 days after the last binge session, with a normal response to cocaine-induced CPP and an increased response in cocaine SA [32]. In this sense, ours and other studies suggest that withdrawal from a high-fat diet can induce cross-sensitization behavior with drugs of abuse.

The aim of the present study was to evaluate if the previously reported effects of HFB are long lasting and remain after fat discontinuation. To this end, mice were exposed to a HFB pattern during adolescence and EtOH self-administration (SA), conditioned place preference (CPP) and locomotor sensitization were evaluated 2 weeks after cessation of this diet. We employed the limited access model of Corwin and co-workers [41], in which animals escalate their intake of a high-fat diet (binge) with intermittent (Monday, Wednesday, Friday) and limited (2h) access. These animals are satiated and develop a binge-eating pattern with palatable food, as they have ad libitum access to standard chow and limited access to high-fat food. Although few studies of high-fat diets have been published, we hypothesized that, based on previous results on cocaine [32], animals that binge on fat would continue to be more vulnerable to the rewarding effects of ethanol after a period of fat discontinuation.

## Materials and methods

### Subjects

A total of 116 male mice of the OF1 strain were acquired commercially from Charles River (France). Animals were 21 days old on arrival at the laboratory and were all housed under standard conditions in groups of 4 (cage size 28 x 28 x1 4.5cm) for 5 days prior to initiating the experimental feeding condition, at a constant temperature (21±2°C), lights on from 8:00 to 20:00, and food and water available *ad libitum* (except during the behavioral tests). All procedures involving mice and their care complied with national, regional and local laws and regulations, which are in accordance with Directive 2010/63/EU of the European Parliament and the council of September 22, 2010 on the protection of animals used for scientific purposes. The Animal Use and Care Committee of the University of Valencia approved the study.

### Drugs

For the oral self-administration procedure, absolute ethanol (Merck, Madrid, Spain) was dissolved in water using a w/v percentage; i.e. a 6% (w/v) ethanol solution equivalent to a 7.6% (v/v) ethanol solution. Saccharin sodium salt (Sigma, Madrid, Spain) was diluted in water. Ethanol (Scharlab S.L., Barcelona, Spain; EtOH) obtained from an initial stock of a 96% v/v solution was diluted at a concentration of 20% v/v in physiological saline (NaCl 0.9% w/v; Sal) and injected intraperitoneally (IP) at the dose of 0.75 and 2 g/kg for the CPP and the locomotor activity experiments, respectively. Control mice were injected IP with the corresponding volume of Sal.

### Apparatus and procedure

#### Experimental design

We employed 4 different sets of animals in this study and performed a single experimental procedure. Each set was composed of 2 groups: control and HFB. One set performed the SA procedure, the second set underwent the CPP, a third set carried out the locomotor sensitization test, and a fourth set was employed for plasma corticosterone determinations.

Control mice in each set were fed a standard diet in their cages and during the binge sessions. Animals in the HFB condition received the standard diet in their home cages but had 2h access every Monday, Wednesday and Friday (MWF) to high-fat food until 15 days before the behavioral tests (HFB). During the SA procedure, access to standard food was restricted to only 1h per day and the HFB group ceased bingeing. The food restriction schedule produced weight loss in mice of around 15% with respect to their free-feeding weights [42]. The remaining sets were not deprived of food during the procedure.

**Fig 1:**
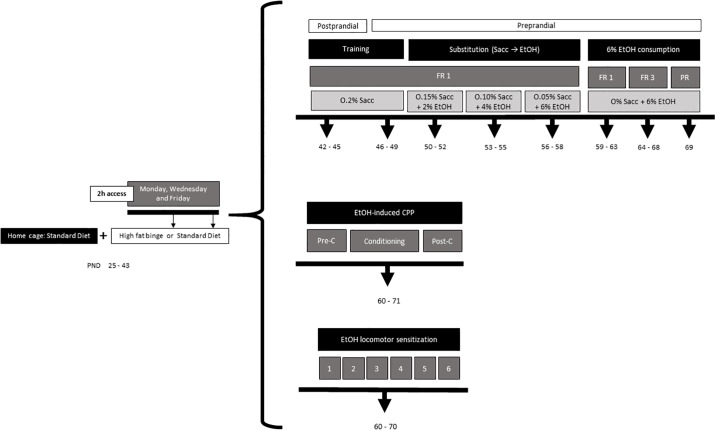
Experimental design.

A thorough description of the experimental procedure is shown in Fig 1.

#### Feeding conditions

The feeding procedure is based on the Limited Access Model described by Corwin et al. [41], in which non-food deprived animals with sporadic limited access to high-fat food develop binge-type behaviors. Two different types of diet were used in this study. The control group was fed with the standard diet (Teklad Global Diet 2014, 13 kcal % fat, 67 kcal % carbohydrates and 20% kcal protein; 2,9kcal/g) and the high-fat diet binge group with a high fat diet (TD.06415, 45 kcal % fat, 36 kcal % carbohydrates and 19% kcal protein; 4,6 kcal/g). The different diets were supplied by Harlan Laboratories Models, S. L. (Barcelona, Spain) and will be referred to from this point onward as the standard diet and the high-fat diet, while the sporadic limited access to high-fat food will be referred to as high-fat diet binge (HFB).

Mice were acclimatized for 5 days before initiating experiments. They were randomly divided into two groups with similar average bodyweight (25-26g) and assigned to either a Control (C) diet or a high-fat diet binge with 15 days withdrawal (HFB), which consisted in 2h access on MWF. All groups were fed the standard diet in their own cages and were allowed to binge for 2h, 3 days a week, in a separate plastic cage, the control group a standard diet, and the HFB group a high-fat diet. Water was freely available at all times.

Binge sessions started on PND 25 and finished on PND 39, taking place every MWF for 2h. Animals began the Training Phase on PND 42 and the 6% EtOH consumption phase on PND 59. The escalation in consumption of the high-fat diet from the first week of access (PND 25–29) and until last week before the tests (PND 39–43) was significant and therefore confirmed a binge-eating pattern. Mice and the food (standard diet) in their home cage were weighed every MWF throughout the study.

#### Oral ethanol self-administration

This procedure is based on that employed by Navarrete et al. [43]. The set was composed of animals fed a standard diet during the binge sessions (n = 10) and animals in the HFB condition (n = 20) allowed 2h access every MWF to high-fat food until 15 days before the 6% EtOH consumption phase.

Oral ethanol self-administration was carried out in 5 modular operant chambers (Med Associates, Inc.) and Med-PC IV software controlled stimuli and fluid delivery and recorded operant responses. These chambers had two small holes with adjacent photocells to detect nose-poke responses. Active nose-poke delivered 37 μl of fluid combined with a 0.5s stimulus light and a 0.5s buzzer beep, followed by a 6s time out period. Inactive nose-pokes did not have any consequence.

The experiment was carried out in three phases: training, saccharin substitution and 6% EtOH consumption.

Training phase (8 days): Two days before beginning the experiment, access to the standard diet was restricted to only 1h per day. Before the first training session, water was withheld for 24h, and food was provided 1h prior to the 1h session to increase the animals’ motivation for lever pressing. During the subsequent 3 days, water was provided ad libitum except during food access, for 1h before beginning each session, in which the water bottle was removed from the cages (postprandial). The following four days and during the rest of the experiment, access to food was provided for 1h after the end of each daily session and water was available ad libitum to avoid EtOH consumption due to thirst (preprandial). The food restriction schedule produced weight loss in mice of around 15% of their free-feeding weights [42]. Mice were trained to nose-poke to receive 37 μl of 0.2% (w/v) saccharin reinforcement.

Saccharin substitution (9 days): The saccharin concentration was gradually decreased as the EtOH concentration was gradually increased [44, 45]. Each solution combination was set up to three consecutive sessions per combination (0.15% Sac -2% EtOH; 0.10% Sac -4% EtOH; 0.05% Sac -6% EtOH).

6% ethanol consumption (11 days): The aim of the last phase of the experimental procedure is to evaluate the number of active nose-poke responses, and 6% EtOH (w/v) intake and the motivation to drink it. To achieve this goal, during the last phase, the number of effective responses and EtOH consumption (g/kg) were measured under fixed ratio 1 (FR1) for 5 daily consecutive sessions, fixed ratio 3 (FR3) (mice had to respond three times to the active nose-poke to achieve one reinforcement) for 5 daily consecutive sessions. After each session, the EtOH remaining in the dispenser was collected with a syringe and this quantity was subtracted from the total amount of EtOH. Finally, on the subsequent day to FR3, a progressive ratio (PR) session was carried out to establish the breaking point for each animal (the maximum number of nose-pokes performed by an animal to earn one reinforcement). The response requirement to achieve reinforcements escalated according to the following series: 1-2-3-5-12-18-27-40-60-90-135-200-300-450-675-1000. To evaluate motivation toward EtOH consumption, the breaking point was calculated as the maximum number of consecutive responses an animal performed to achieve one reinforcement, according to the previous scale (for example, if an animal nose-poked a total of 108 times, it was considered to have responded a maximum of 40 consecutive times for one reinforcement. Therefore, the breaking point value for this animal would be 40). All the sessions lasted 1h, except the PR session, which lasted 2h.

#### Conditioned place preference

For place conditioning, 8 identical Plexiglas boxes with two equally sized compartments (30.7 cm long × 31.5 cm wide × 34.5 cm high) separated by a grey central area (13.8 long × 31.5 cm wide × 34.5 high) were employed. The compartments had different colored walls (black vs. white) and distinct floor textures (smooth in the black compartment and rough in the white one). Four infrared light beams in each compartment and six in the central area allowed the recording of the position of the animal and its crossings from one compartment to the other. The equipment was controlled by two IBM PC computers using MONPRE 2Z software (CIBERTEC, SA, Spain).

To evaluate the consequences of HFB withdrawal on the acquisition of EtOH-induced CPP (n = 15 per group), animals were subjected to an unbiased CPP procedure consisting of three different phases: preconditioning (1 session; Pre-C), conditioning (8 sessions), and preference testing (1 session; postconditioning, Post-C). In the first day (Pre-C), mice were placed in the central area of the apparatus with the guillotine doors opened to give them access to both compartments of the apparatus for 900s. The time spent by the animal on each side during this period was recorded. Subjects showing a strong unconditioned aversion (<33% of the session time) or preference (>67%) for any cubicle were discarded from the study. The conditioning phase began 24h after Pre-C. In each group, half of the animals received EtOH in one compartment and the other half in the other in a counterbalanced manner. After randomly assigning the animals to a conditioning chamber, an ANOVA confirmed that there were no significant differences between the time spent in the EtOH- and the Sal-paired compartments during Pre-C. In the second phase (conditioning), half of the mice were injected with Sal and the other half with EtOH (0.75 g/kg) and were placed immediately in their corresponding conditioning chamber. On alternate days, the contingencies were inverted and the animals that had received Sal the day before were injected with EtOH (0.75 g/kg) and those who had received EtOH (0.75 g/kg) were given Sal just prior to introducing them into the other conditioning compartment. During an 8-day period subjects received a total of 4 pairings for each condition, each pairing separated by a 24h interval. The central area was made inaccessible during the 5-min conditioning trial by closing the guillotine doors. The duration of the EtOH pairing was selected because it has been shown to be sufficient to induce CPP in mice [46, 47]. We decided to administer the dose of 0.75 g/kg because pilot studies in our laboratory and previous published reports have shown that doses of EtOH below 1 g/kg are unable to produce CPP in standard mice [48]. The preference test (Post-C) took place 24h after the last conditioning assay. During Post-C, the guillotine doors separating the two cubicles were removed and the time spent by the untreated mice in each chamber was recorded for 900s.

#### EtOH-induced locomotor sensitization

To assess the effect of HFB withdrawal on the locomotor sensitization elicited by EtOH (2 g/kg), animals (n = 10 per subgroup) were tested in open-field chambers that consisted of four Plexiglas cages (30 cm long × 30 cm wide × 35 cm high) in which locomotor activity was registered by a computerized video-tracking system (Ethovision, Noldus S.A., The Netherlands). Movement of the mouse inside the open-field chambers was recorded and translated automatically by the software to horizontal distance traveled in cm over 10 min. On the habituation day, mice were allowed to spend 10 minutes expelling the chambers without drug in order to eliminate the novelty effects and set a locomotor baseline. The sensitization training protocol involved six trials: one trial per day on alternate days. For the experiment, mice were taken from the vivarium and brought to the experimental chamber 10 min prior to the session. At the start of each assay, subjects were given an injection of Sal or EtOH (2 g/kg) and immediately placed in the center of the activity enclosure for 10 min. This procedure was selected based on previous reports showing that it is able to evoke locomotor sensitization in mice [49].

#### Determination of plasma corticosterone

The fourth set was employed for plasma corticosterone determinations (n = 8 per group) on PND 58, 15 days after the last binge session, in order to confirm a withdrawal state. Blood samples were taken by means of the tail-nick procedure, in which the animal is wrapped in a cloth and a 2-mm incision is made at the end of the tail artery. The tail is then massaged until 50 μl of blood is collected in an ice-cold Microvette^®^ CB 300 capillary tube (Sarstedt, Germany). Blood samples were kept on ice, and plasma was separated from whole blood by centrifugation (5 min, 5000 g) and transferred to sterile 2 ml microcentrifuge tubes. Plasma corticosterone levels were measured with an ELISA kit from Enzo^®^ Life Sciences (Catalog No. ADI-900-097), following the manufacturer’s instructions. The sensitivity of the test is 0.2. All samples were run in duplicate.

#### Blood ethanol concentration

On day 4 (FR1), after the 1 h self-administration session, blood was collected from both groups to assess blood ethanol concentration (BEC) Blood sampling determination was performed by the tail-nick procedure, as previously described. The supernatant was then placed in cuvettes with optical properties suitable for use with a spectrophotometer set at 340 nm. Blood ethanol content was enzymatically determined with the NAD-ADH Reagent Multiple Test Vial Kit (Sigma Aldrich S.A.).

### Statistics

Data relating to body weight and intake during bingeing were analyzed by a one-way ANOVA with a within variable PND with 13 levels (PND 25 to 69) in the case of bodyweight and 7 levels for the binge sessions: PND 25, 27, 29, 32, 34, 36, 39; in which animals were weighed and commenced the binge session.

To analyze acquisition of EtOH SA, a two-way ANOVA was performed with Diet (control or HFB) as a between factor and Days (5 levels for FR1 or FR3) as a within factor. A Student’s t-test was employed to analyze breaking point values and ethanol consumption during PR. For the CPP, the time spent in the drug-paired compartment was analyzed by means of a mixed analysis of variance (ANOVA) with one between variable: Diet, with 2 levels (Control, HFB). Data from the horizontal locomotion (cm) carried out during the first and the sixth days of treatment were analyzed by means of a three-way ANOVA with Diet (Control or HFB) and Treatment (Saline or EtOH) as the between-subjects variables and Days (1 and 6) as the within-subjects variable. The corticosterone and BEC data were analyzed by means of Student’s t-tests. Subsequent Bonferroni post—hoc tests were calculated when required. Data are presented as mean ± SEM. Analyses were performed using SPSS v22.

## Results

### Body weight and binge escalation

**Fig 2:**
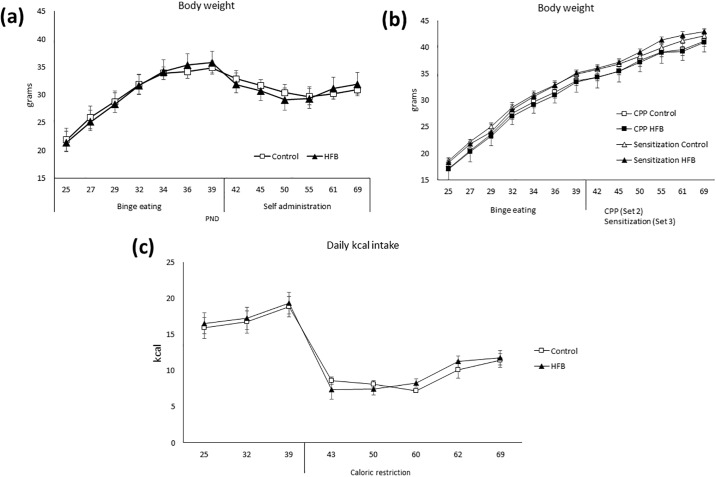
(a) Bodyweight of the first set of animals (Self-administration) during the whole procedure. (b) Bodyweight in the second and third set of animals during the whole procedure (c) Individual mean daily intake in the first set of animals. Phase 1: PND 25–42; Phase 2: PND 43–71: food restriction to 1h/day.

The ANOVA of the body weight (Fig 2a) revealed an effect of the variable Day [F(12,336) = 374.951; p<0.001] and the interaction Days x Diet [F(12,336) = 4,995; p<0.001]. The bodyweight of all the animals increased during the experiment (p<0.001, with respect PND 25). During the SA procedure, when access to food was restricted, all animals lost approximately 15% of their body weight. Sets 2 and 3 (Fig 2b) presented similar bodyweight to set 1 until the beginning of the SA procedure, when the bodyweight of Set 1 decreased to 85% of their original weight (Fig 2a). No significant differences in body weight were detected between the control and HFB groups during the course of the experiment.

**Fig 3:**
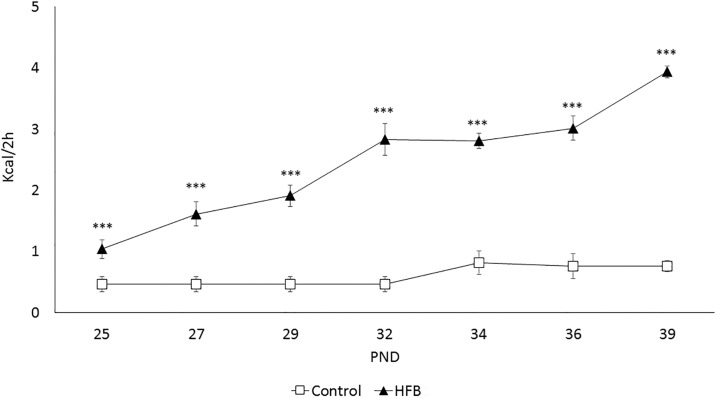
Intake (kcal) during the high-fat binge-eating sessions that took place on MWF (2h access in all groups). The mean (± SEM) amount of kcal consumed in 2 hours of limited access to high-fat food (control group had access to standard food) is represented to confirm the escalation of intake. ***p<0.001 significant difference with respect to the control group.

Confirming the binge pattern of intake of a high-fat diet, the consumption of all animals escalated in every session (Fig 3), as the ANOVA showed an effect of the variable Diet [F(1,28) = 115,247; p<0.001]. Mice in the HFB showed a higher intake of high-fat food during the binge sessions with respect to the control group. Likewise, our results revealed a significant difference of the variable Days [F(6,168) = 20,333; p<0.001] and the interaction Days x Diet [F(6.168) = 12,419; p<0.001]. The HFB showed an escalation in the intake of high-fat food kcal from day 1 onwards (p<0.001 all days).

### Oral ethanol self-administration

No differences were found between animals during the training phase, showing that bingeing on fat did not induce any learning deficit (S1 Table).

**Fig 4:**
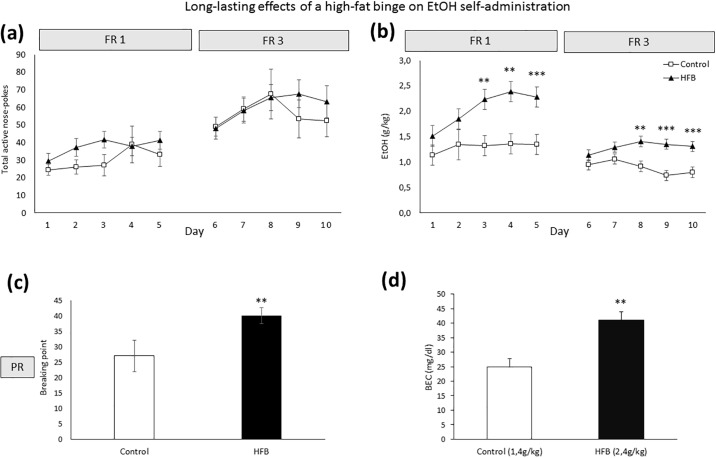
Analysis of oral EtOH self-administration in OF1 mice (n = 30). The dots represent means and the vertical lines ± SEM of (a) the number of effective responses and (b) amount of 6% EtOH consumption during FR1 and FR3 (in g/kg). The columns represent means and the vertical lines ± SEM of (c) breaking point values during PR and (d) blood ethanol concentrations on day 4 (FR1) **p<0.01; ***p<0.001 values for HFB mice that are significantly different from those of control mice.

With respect to the long-lasting effects of a HFB (See Fig 4), the ANOVA of the FR1 schedule revealed a significant effect of the variable Days on the effective number of responses [F(4,100) = 4,018 p<0.01] and EtOH consumption (g/kg) [F(4,100) = 6,190 p<0.001] (Fig 4a and 4b), with significant differences on day 1 compared to day 3 (p<0.01), 4 (p<0.01) and 5 (p<0.05). There was also an effect of the variable Diet [F(1,25) = 7,336; p<0.01] and the interaction Diet x Days [F(4,100) = 2.603 p<0.05] on EtOH consumption. Mice in the HFB group exhibited an increased oral SA of EtOH (6%) with respect to the control group (Fig 4b) on days 3 (p<0.01) 4 (p<0.01) and 5 (p<0.001).

The ANOVA of the FR3 schedule revealed a significant effect of the variable Days on effective responses [F(4,100) = 3,114 p<0.05] and indicated a significant effect of the interaction Days x Diet [F(4,100) = 2.946; p<0.05] on EtOH consumption. Bonferroni post-hoc analyses showed higher EtOH intake in the HFB group on days 3, 4 and 5 (p<0.01 on day 3 and p<0.001 on days 4 and 5).

Analyses of the PR showed an effect of the variable Diet [F(1,23) = 5,938 p<0.05). Breaking point values were significantly higher among animals in the HFB group (Fig 4c).

Finally, there was a significant effect on BECs (Fig 4d), as animals in the HFB group achieved higher BEC during the session with respect to the control group (Student’s t-test, t = -3,853, 10 d.f.; p<0.01).

### Effects of a HFB until 15 days before the acquisition of EtOH-induced CPP

**Fig 5:**
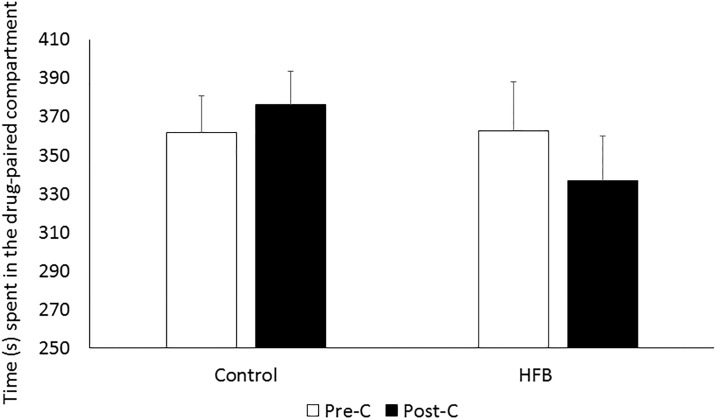
Effects of a HFB until 15 days before the acquisition of 0.75g/kg EtOH-induced CPP. Bars represent mean (±SEM) of time spent in the EtOH-paired compartment for the different Diet (C or HFB) groups (n = 15 per group) during Pre-C (white bars) and Post-C (black bars).

As can be seen in Fig 5, the HFB did not have an effect on the conditioning properties of EtOH (0.75 g/kg). The time spent in the EtOH-paired chamber on Post-C day did not differ from that of Pre-C in any of the groups.

### Effects of HFB until 15 days before the EtOH-induced locomotor sensitization

**Fig 6:**
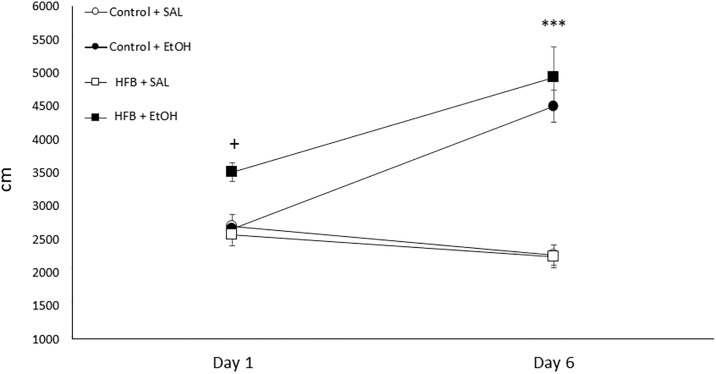
Effects of bingeing intermittently on a high-fat diet until 15 days before EtOH-induced locomotor sensitization. Values represent mean (±SEM) of locomotor activity (cm in 10 min) for mice (n = 10 per subgroup) previously exposed to a standard or HFB diet and treated with Sal or EtOH (2 g/kg) immediately before being introduced into the open field on 6 alternate days. *** p<0.001 significantly different from Day 1; + p<0.05 significantly different from Sal on day 1.

Fig 6 displays the locomotor-sensitizing effects of EtOH. The ANOVA indicated a significant effect of Treatment [*F*(1, 36) = 39.096, *p*<0.001] and Days [*F*(1,36) = 28.050, *p<*0.001], and the interaction Days × Treatment [*F*(1,36) = 73.239, *p*<0.001]. The post-hoc test revealed that EtOH-induced sensitization occurred in both conditions, since the locomotor activity of both groups significantly increased on day 6 with respect to day 1 (*p*<0.001). In addition, the acute locomotor activity response to EtOH on day 1 was significantly higher in the HFB group than in the rest of the groups (*p<*0.05).

### Effects of high-fat diet discontinuation on circulating corticosterone levels

**Fig 7:**
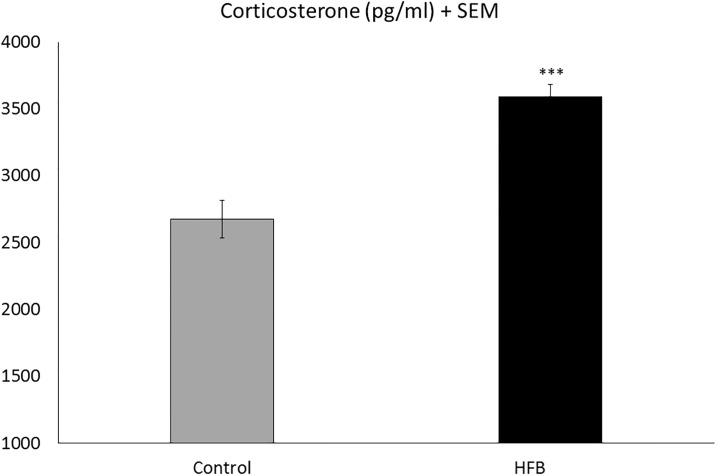
Effects of HFB discontinuation on circulating corticosterone levels. Data are presented as mean values ± S.E.M. (pg/ml) ***p<0.001 with respect the control group.

Corticosterone analyses indicated an increase in plasma corticosterone levels in animals in the HFB group with respect to the control group (Student’s t-test, t = -5.831, 14 d.f.; p<0.001) (see Fig 7).

## Discussion

The current study shows for the first time that bingeing on fat during adolescence induces long-lasting consequences on the rewarding effects of EtOH. We have assessed how animals that binged intermittently on fat during adolescence and abstained from fat 2 weeks before the beginning of the behavioral procedures consumed higher quantities of EtOH in the oral SA paradigm and showed an increased motivation to obtain the drug in the progressive ratio schedule. Although mice exposed to HFB were not more sensitive to EtOH-induced CPP, they still presented a higher motor response to this drug.

In a previous report [33], we found that bingeing on fat during adolescence increased the rewarding effects of EtOH while the animals continue to binge. Animals bingeing on fat showed greater EtOH consumption and a higher motivation to obtain the drug. HFB mice also developed preference for the paired compartment in the CPP with a subthreshold dose of EtOH. In the present study, we have confirmed that the increase in EtOH consumption is long lasting after fat consumption has ended.

Regarding the oral SA, under the FR1 and FR3 schedules HFB mice exhibited an increase in EtOH consumption and a higher number of effective responses with respect to the control group. Both groups similarly acquired and maintained a stable operant response, therefore discarding a possible learning deficit. The HFB group exhibited an escalation in EtOH consumption as time passed, both in FR1 and in FR3, which may have been due to the absence of the other reinforcer, since motivation is enhanced after a period of abstinence [50]. The results of blood ethanol concentrations are in the line of those of previous studies in which 25-40mg/dl were considered adequate blood ethanol levels after oral self-administration in standard animals [51–53]. We also observed a higher progressive ratio response in HFB mice, which demonstrates an increased motivation and compulsivity to obtain the drug. These results are in line with those of Avena and colleagues [34], who used food rich in sugar. They reported that rats allowed intermittent access to sugar for 21 days and subsequently blocked for 3 days consumed more EtOH in a two-bottle choice paradigm. In a recent study by Sirohi and co-workers [54], adult rats intermittently administrated HFD for 5 weeks displayed attenuated acquisition of alcohol intake (20%). Differences in the age at which animals were exposed to the high-fat diet (adult vs adolescent) and the procedure employed to evaluate EtOH consumption may explain discrepancies with our results.

However, we did not observe any difference regarding the conditioned rewarding effects of a subthreshold dose of EtOH like 0.75g/kg on the CPP paradigm. Previous studies point to the fact that doses below 1g/kg are unable to produce CPP in standard mice [48]. Although we have observed an increase in the conditioned rewarding effects of EtOH when mice binge on fat [33], no effect was detected 2 weeks after the last binge. Similar results were obtained in one of previous studies with cocaine [32], in which neither of our withdrawal groups responded to a subthreshold cocaine dose in the CPP, as occurred with EtOH in the present work. Nevertheless, when an effective dose was administered, HFB withdrawal groups were more resistant to the extinction of memories associated with reward. However, in the cocaine SA study, re-exposure to fat after a period of abstinence significantly increased the number of active nose-pokes, showing a reinstatement into drug-seeking. The CPP procedure aims to evaluate the relevance of the environmental cues associated with the drug [55], based on the change from initially neutral environmental cues to conditioned stimuli with secondary motivational properties [56]. On the other hand, the SA paradigm evaluates the direct primary reinforcing effects of drugs according to the effort made by the animal to obtain the drug. Fifteen days after cessation of bingeing on fat, our mice showed normalized development of the conditioned environmental cues, while the primary reinforcing properties of EtOH were still increased.

Although it can be argued that the animals in the HFD group ingested more ethanol for its caloric effect, the lack of differences in the Kcal consumption of the standard diet would question this. In addition, our data are in accordance with those regarding the effects of sugar-rich diets, in which animals forced to abstain from sucrose have been shown to present an enhanced response to ethanol, methamphetamine and cocaine [34, 57, 58].

Behavioral sensitization has been considered one of the key components in drug addiction [59]. Two weeks after bingeing on fat during adolescence, we observed the development of sensitization to the locomotor effects of EtOH in controls and mice exposed to fat. However, the acute locomotor response to EtOH in the HFB group on day 1 was enhanced with respect to the control group. This result is similar to that observed in mice while bingeing on fat during the whole procedure [33]. In the same line, other studies have already described the increased locomotor effects of psychostimulants in animals fed a high-fat diet [7, 60].

These long-lasting effects of fat bingeing can be explained by several mechanisms. Withdrawal from access to a cafeteria or high-fat diet has been shown to induce an abnormal function of the HPA axis with a significant increase in CRF and basal corticosterone levels [31, 61–63]. Our results show an increase in corticosterone levels after fat discontinuation, similarly to that reported by other studies [32, 64, 65]. Therefore, we can speculate that animals in a state of fat-binge withdrawal experience an aversive emotional state after cessation of palatable food and seek to compensate this lack of pleasure with other rewards, such as ethanol.

On the other hand, bingeing on fat could induce neuroadaptations that remain after fat binge eating interruption. We have previously detected modifications in gene expression of the dopaminergic, opioid and endocannabinoid systems after exposure to a HFB. A similar exposure to HFB as that in the present study induces a decrease in gene expression of the CB1 receptor and mu opioid receptor in the nucleus accumbens (N Acc) [32]. In the subsequent study, we found that some of these gene expression changes persisted after exposure to HFB and EtOH consumption, such as the reduction in the mu opioid receptor in the N Acc; however, in contrast with previous results, CB1r gene expression increased significantly [33]. There is a known modulatory action between the opioid and cannabinoid systems [66, 67]. While mu opioid receptor activation is vital for intake of fat and EtOH [68, 69], CB1 receptors are critical for emotional and motivational responses [33, 70].

Some neuroadaptations induced by HFD could persist after the cessation of ingestion of fat, highlighting the long-lasting effect of this diet on the reward system. For example, regarding changes in the dopaminergic system, we have previously found that Tyrosine Hydroxylase (TH), which is involved in the synthesis of dopamine, is decreased in the VTA after EtOH consumption in mice exposed to a fat-binge, which could represent a compensatory action to chronic overstimulation of the mesolimbic pathway [33]. In line with this, a recent study by Carlin and co-workers [71] found that female mice exposed during adolescence to a high-fat diet showed an increase in dopamine transporter expression and a decreased tyrosine hydroxylase expression in the VTA. In addition, nutritional manipulation by means of a high-fat diet has been shown to affect the long-term functioning of the DA system in rodents [72]. Teegarden and Bale reported that withdrawal from a HFD could induce deep alterations of the reward systems, such as a reduction in the dopaminergic signal in mice previously exposed to a HFD and abstaining from fat for 24 h, 48 h, and 1 week [61].

In summary, our results indicate that intermittent access to high-fat food can induce long-lasting changes in the brain that lead to an increased consumption of EtOH and vulnerability to its reinforcing properties. The results of the current study might have clinical implications, as they suggest that even when adolescents interrupt their binge-eating, they continue to be more vulnerable to EtOH consumption than their non-bingeing peers.

## Supporting information

S1 Table Training and saccharin substitution phases. (XLSX)
